# Recreational Drug Use among Chinese MSM and Transgender Individuals: Results from a National Online Cross-Sectional Study

**DOI:** 10.1371/journal.pone.0170024

**Published:** 2017-01-20

**Authors:** Peizhen Zhao, Songyuan Tang, Cheng Wang, Ye Zhang, John Best, Thitikarn May Tangthanasup, Shujie Huang, Bin Yang, Chongyi Wei, Joseph D. Tucker, Weiming Tang

**Affiliations:** 1 Guangdong Center for Skin Diseases and STI Control, Guangzhou, China; 2 University of North Carolina at Chapel Hill Project-China, Guangzhou, China; 3 SESH study group of University of North Carolina at Chapel Hill, Guangzhou, China; 4 Kunming Medical University, Kunming, China; 5 School of Medicine, University of California, San Francisco, San Francisco, United States of America; 6 School of Medicine of University of North Carolina at Chapel Hill, Chapel Hill, United States of America; Fudan University, CHINA

## Abstract

**Background:**

Recreational drug use has increased considerably among Chinese men who have sex with men (MSM). The phenomenon has the potentially to increase HIV transmission among Chinese MSM. The aims of this study were: 1) to investigate the prevalence of recreational drug use among Chinese MSM, and 2) to explore the correlation between gay smartphone based sex-seeking applications (gay apps), HIV/STIs testing, group sex, commercial sex, sexual roles and poppers use among Chinese MSM.

**Methods:**

MSM who were born biologically male, were at least 16 years of age and had engaged in anal sex with a man at least once were recruited through a nation-wide online survey in 2014. Information regarding socio-demographics, risk behaviors, recreational drug use, HIV and other STIs testing history and gay app use were collected. Univariate and multivariate analysis were used to determine factors associated with recreational drug use among Chinese MSM.

**Results:**

Among 1424 participating MSM, 1100 (77.3%) reported ever using recreational drugs in their lifetime. In the last 12 months, 303 (21.3%) used poppers, 34 (2.4%) used crystal meth and 15 (1.0%) used ecstasy. The mean age of respondents was 25.6±6.8 years, 72.9% identified as gay, 41.3% were students, and 83.8% had never been married. Multiple logistic regression models revealed that compared with non-popper users, popper users were more likely to have been tested for HIV (adjusted OR (aOR) = 1.50, 95% CI: 1.15–1.96) and other STIs (aOR = 1.65, 95% CI: 1.26–2.17). In addition, popper users were more likely to engage in group sex (aOR = 2.63, 95% CI:1.80–3.86), commercial sex (aOR = 1.86, 95% CI:1.13–3.06) and used gay mobile apps to seek sexual partners (aOR = 2.10, 95% CI:1.58–2.80).

**Conclusion:**

Chinese MSM has a high rate of recreational drug use, including poppers. Public health programs serving MSM may consider integrating intervention programs to decrease recreational drug use related harms.

## Introduction

Recreational drug use is long being considered to a major global driving force for HIV transmission[1]. With the implementation of harm reduction programs, however, HIV incidence rates have decreased among people who inject drugs around the world[2–4]. Despite these achievements in HIV transmission prevention among people who inject drugs, many challenges still remain [5]. In addition, new challenges have emerged in the last decade due to increasing prevalence of recreational drug use among men who have sex with men (MSM) and transgender individuals [6].

Recreational drug use is not uncommon among MSM, and may be associated with risk of HIV and sexually transmitted infections[7]. Recreational drug use and subsequent states of intoxication may influence sexual risk behavior among MSM [6]. Recreational drug use affects HIV transmission via several mechanisms, including physiological responses, routes of administration, venues at which drugs are used, incidence of HIV infection in specific drug-using populations, and cognitive effects on decision making [8]. Under the effect of recreational drugs, MSM may to have multiple sexual partnerships [9], participate in group sex[10], engage in condomless anal intercourse [11] and experience more intimate partner violence [12], which may further facilitate HIV transmission.

Although a number of studies have examined the association between recreational drug use and risk behaviors for HIV transmission, its association with HIV and other STIs testing history, smartphone-based sex-seeking applications (gay apps) use, and sexual role during sex have not been well documented. China presents a unique opportunity to examine these correlations. In China, recreational drug use has increased considerably among MSM in recent years [13], and an alarmingly high rate of recreational drug use has been observed among Chinese MSM[14]. In addition, gay app use has been relatively widespread [15] and rapidly adopted [16] among Chinese MSM. Smartphone use may foster virtual risk environments for HIV transmission among Chinese MSM, as the gay app use has the potential to facilitate the organization of private sex parties involving recreational drug use[17].

The main aim of this study was to characterize the proportion of different types of recreational drug use among MSM in China at a national level. In addition, we aimed to explore the association between popper (the most common recreational drug in China) use and other novel influencing factors (HIV and other STIs testing history, gay apps use, and sexual role during sex among Chinese MSM).

## Methods

### Study design and sampling methods

A cross-sectional nation-wide online survey was conducted between September and October of 2014.

In the survey, MSM across China were recruited through three gay web portals: Northern China (http://www.danlan.org), Southern China (http://www.yntz.net), and Eastern China (http://www.jstz.org). Web portals serve as an especially common online entry points for a number of different services, such as exchanging news, social networking, finding sex partners, and advertising gay specific products and research. Detailed description of sampling strategy and recruitment has been reported elsewhere [18]. We followed a checklist for reporting results of Internet e-surveys (CHERRIES) throughout the process to improve the quality and reporting of our web survey[19].

In order to recruit participants, the banner links of the survey were displayed on the homepages of the aforementioned web portals. In addition, we also sent a survey introduction with a survey link to registered users of the three web portals. After clicking the banner links, interested participants were directed to the online survey. To be eligible, participants must meet the following criteria: being born as male, at least 16 years old, had ever engaged in anal sex with another man, willing to provide their cell phone number (for other follow- up purposes) and agreed to an informed consent.

### Measures

After meeting criteria and signing the consent form, participants provided socio-demographic information: age (continuous, and was further categorized into three groups: less than 20, 20–29, or 30 and above), occupation (student or not), marital status (never married or ever married, including widowed or divorced), education (senior high school or below, college/bachelors, or masters or PhD), and annual income (less than 3000 USD, 3000–6000 USD, 3001–9600 USD, 9601–15000 USD or above 15000USD). In addition, we also collected information on their sexual orientation (gay or bisexual) and their current self-identified gender (male or transgender). We also collected information behaviors such as HIV and other STIs testing history (yes or no), whether or not participants currently have a main partner (yes or no), their preferred sexual role during anal sex (insertive, receptive or no preference), history of vaginal or oral sex with women (yes or no), whether or not they had condomless sex with female partners in the last three months (yes or no), whether or not they had condomless sex with male partners in the last three months (yes or no), ever drunk alcohol during or prior sex in the last three months (yes or no), whether or not they had participated in group sex in the last 12 months (yes or no), engaged in commercial sex in the last 12 months, and whether or not they had used smartphone-based sex-seeking applications (gay apps) in the last six months.

Every participant was also asked whether or not they had used any of the listed recreational drugs [poppers, ecstasy, crystal meth, or others] prior to sex in the last 12 months.

### Statistical analysis

Descriptive analysis was performed to describe socio-demographics, risk behaviors, and recreational drug use among participants who used poppers in the last 12 months compared to those and who did not. Univariate and multivariate logistic regressions were used to evaluate factors associated with poppers use (the most popular recreational drug) among Chinese MSM. In this study, demographic characteristics, including age, residence, education, marital status, and income were adjusted for in the multivariate logistic regression models. All data were analyzed by using SAS 9.4 (SAS int. Cary, NC, USA).

### Ethical statement

Ethical approval was obtained from institution review committees in China (Guangdong Provincial Center for Skin Diseases and STI Control), and the United States (University of North Carolina at Chapel Hill and the University of California, San Francisco (14–1865)). The study protocol as well as the inform consent were all approved by the ethics committees. All participants agreed to an informed consent. Written consent from was obtained from each participant prior to the survey, while informed consents from the next of kin, caretakers, or guardians on behalf of the minors/children enrolled in the study was not obtained. The anonymous data (S1 Dataset) and written inform consents were kept electrically and confidential.

## Results

### Socio-demographic characteristics and behaviors

Altogether 1424 participants were recruited in this cross-sectional survey. The mean age of participants was 25.6±6.8 years. About 63.1% of the participants were aged between 20 and 29 years old, 72.9% of them identified themselves as gay, 68.0% have a college/bachelor and above degree, 41.3% were students, 83.8% had never been married, and 26.0% had annual income less than 3000 USD.

Among 1424 participating MSM, 1100 (77.3%) reported using any recreational drugs in their lifetime. In the last 12 months, 303(21.3%) used poppers, 34(2.4%) used crystal meth and 15(1.0%) used ecstasy.

In our study, a total of 703(49.4%) participants reported that they have been tested for HIV before, 456(32.0%) have been tested for other STIs except HIV, 61(4.3%) were transgender individuals, 691 (48.5%) currently have a main partner, 414(29.1%) of participants reported that they ever had vaginal or oral sex with women. In the last three months, 182(12.8%) of participants had condomless sex with women, 201(14.1%) of them had condomless sex with men and 124 (8.7%) of them had ever drunk alcohol prior to sex. In the last 12 months, 141(9.9%) of participants engaged in group sex, 82(5.8%)of them engaged in commercial sex.

In our study, 824 (57.9%) participants used gay apps for partner seeking in the last six months. About 72.6% of the poppers users and 53.9% of the non-poppers users used gay apps for partner seeking in the last six months, respectively.

Detailed information regarding socio-demographics, behaviors for both poppers users and non- poppers users are also presented in Table 1.

**Table 1:** Demographic characteristics, behaviors and HIV/other STIs testing history among men who have sex with men in China, 2014 (N = 1424).

Variables		Poppers users(n = 303)			Non users(n = 1121)			Overall	
		Frequency	Percent	95% CI	Frequency	Percent	95% CI	Frequency	Percent
Age group	*Less than 20*	36	11.9	8.2,15.5	170	15.2	13.1,17.3	206	14.5
	*20 to 29*	203	67.0	61.7,72.3	695	62.0	59.2,64.8	898	63.1
	*30 or above*	64	21.1	16.5,25.7	256	22.8	20.4,25.3	320	22.5
Sexual orientation	*Gay*	234	77.2	72.5,82.0	804	71.7	69.1,74.4	1038	72.9
	*Bisexual*	69	22.8	18.0,27.5	317	28.3	25.6,30.9	386	27.1
Education	*Senior high school or below*	66	21.8	17.1,26.4	303	27.0	24.4,29.6	369	25.9
	*College / Bachelors*	219	72.3	67.2,77.3	750	66.9	64.1,69.7	969	68.0
	*Masters or PhD*	18	5.9	3.3,8.6	68	6.1	4.7,7.5	86	6.0
Marital status	*Never married*	265	87.5	83.7,91.2	929	82.9	80.7,85.1	1194	83.8
	*Ever married*	38	12.5	8.8,16.3	192	17.1	14.9,19.3	230	16.2
Student	*Yes*	123	40.6	35.0,46.2	465	41.5	38.6,44.4	588	41.3
	*No*	180	59.4	53.8,65.0	656	58.5	55.6,61.4	836	58.7
Income	*Less than3000 USD*	67	22.1	17.4,26.8	303	27.0	24.4,29.6	370	26.0
	*Between 3000 and 6000USD*	77	25.4	20.5,30.3	343	30.6	27.9,33.3	420	29.5
	*Between 6000 and9600 USD*	90	29.7	24.5,34.9	286	25.5	23.0,28.1	376	26.4
	*Between 9600 and 15000 USD*	46	15.2	11.1,19.2	125	11.2	9.3,13.0	171	12.0
	*More than 15000 USD*	23	7.6	4.6,10.6	64	5.7	4.3,7.1	87	6.1
Ever tested for other STIs except HIV	*Yes*	125	41.3	35.7,46.8	331	29.5	26.8,32.2	456	32.0
	*No*	178	58.7	53.2,64.3	790	70.5	67.8,73.1	968	68.0
Ever tested for HIV	*Yes*	176	58.1	52.5,63.7	527	47.0	44.2,49.9	703	49.4
	*No*	127	41.9	36.3,47.5	594	53.0	50.2,55.9	721	50.6
TG	*Yes*	13	4.3	2.0,6.6	48	4.3	3.1,5.5	61	4.3
	*No*	*290*	*95*.*7*	*93*.*4*,*98*.*0*	*1073*	*95*.*7*	*94*.*5*,*96*.*9*	*1363*	*95*.*7*
Currently have a main partner	*Yes*	159	52.5	46.8,58.1	532	47.5	44.5,50.4	691	48.5
	*No*	144	47.5	41.9,53.2	589	52.5	49.6,55.5	733	51.5
Preferred sexual role during anal sex	*1*	93	30.7	25.5,35.9	431	38.4	35.6,41.3	524	36.8
	*0*	153	50.5	44.8,56.2	468	41.7	38.8,44.6	621	43.6
	*No preference*	57	18.8	14.4,23.	222	19.8	17.5,22.1	279	19.6
Ever had vaginal or oral sex with women	*Yes*	75	24.8	19.9,29.6	339	30.2	27.5,32.9	414	29.1
	*No*	228	75.2	70.4,80.1	782	69.8	67.1,72.4	1010	70.9
Had condomless sex with women in the last three months	*Yes*	31	10.2	6.8,13.7	151	13.5	11.5,15.5	182	12.8
	*No*	272	89.8	86.3,93.2	970	86.5	84.5,88.5	1242	87.2
Had condomless sex with men in the last three months	*Yes*	47	15.5	11.4,19.6	154	13.7	11.7,15.8	201	14.1
	*No*	256	84.5	80.4,88.6	967	86.3	84.2,88.3	1223	85.9
Ever drunk alcohol during or prior sex in the last three months	*Yes*	24	7.9	4.9,11.0	100	8.9	7.2,10.6	124	8.7
	*No*	279	92.1	89.0,95.1	1021	91.1	89.4,92.8	1300	91.3
Participated in group sex in the last 12 months	*Yes*	54	17.8	13.5,22.2	87	7.8	6.2,9.3	141	9.9
	*No*	249	82.2	77.8,86.5	1034	92.2	90.7,93.8	1283	90.1
Changed sex for gifts or money in the last 12 months	*Yes*	26	8.6	5.4,11.8	56	5.0	3.7,6.3	82	5.8
	*No*	277	91.4	88.2,94.6	1065	95.0	93.7,96.3	1342	94.2
Gay app users	*Yes*	220	72.6	67.6,77.6	604	53.9	51.0,56.8	824	57.9
	*No*	83	27.4	22.3,32.4	517	46.1	43.2,49.0	600	42.1
HIV infection	*Positive*	17	5.6	3.0,8.2	51	4.5	3.3,5.8	68	4.8
	*Negative*	145	47.9	42.2,53.5	424	37.8	35.0,40.7	569	40.0
	*Unknown*	141	46.6	40.9,52,2	646	57.6	54.7,60.5	787	55.2

### Correlates of recreational drug use

Univariate analysis indicated that compared to non-users, poppers users were more likely to have been tested for HIV or other STIs except HIV, with crude OR (cOR) of 1.56 (95% CI:1.21–2.02) and 1.68 (95% CI:1.29–2.18), respectively.

Compared to non-users, poppers users were more likely to engage in group sex in the last three months (cOR = 2.58,95% CI:1.79–3.72) and were also more likely engage in commercial sex in the last 12 months (cOR = 1.78, 95% CI:1.10–2.90). Univariate analysis also suggests that poppers users were more likely to use gay apps for partner seeking in the last six months (cOR = 2.27, 95% CI:1.73–3.00).

Similar findings were identified, after adjusted for age, residency, monthly income, education and marital status. For example, compared to non-users, poppers users were more likely to have been tested for HIV (aOR = 1.50, 95% CI:1.15–1.96) or other STIs except HIV (aOR = 1.65, 95% CI:1.26–2.17). Poppers users were also more likely to report engaging in group sex in the last three months (aOR = 2.63, 95% CI:1.80–3.86). These results were shown in Table 2.

**Table 2:** Factors associated with Poppers use among Chinese MSM, 2014 (N = 1424).

Variables	Crude Model		Adjusted Model*	
	OR (95% CI)	*P*-value	OR (95% CI)	*P*-value
**Ever tested for other STIs except HIV**				
*No*	*Ref*		*Ref*	
*Yes*	1.68(1.29,2.18)	<0.001	1.65(1.26,2.17)	<0.001
**Ever tested for HIV**				
*No*	*Ref*		*Ref*	
*Yes*	1.56(1.21,2.02)	<0.001	1.50(1.15,1.96)	0.003
**Student**				
*No*	*Ref*		*Ref*	
*Yes*	0.96(0.74,1.25)	0.78	1.06(0.77,1.46)	0.74
**Transgender**				
*No*	*Ref*		*Ref*	
*Yes*	1.00(0.53,1.87)	0.99	0.94(0.49,1.78)	0.84
**Sexual orientation**				
*Bisexual*	*Ref*		*Ref*	
*Gay*	1.34(0.99,1.80)	0.056	1.28(0.94,1.74)	0.12
**Currently have a main sexual partner**				
*No*	*Ref*		*Ref*	
*Yes*	1.22(0.95,1.58)	0.1213	1.16(0.89,1.50)	0.27
**Preferred sexual role during anal sex**				
*1*	*Ref*		*Ref*	
*0*	1.52(1.14,2.02)	0.005	1.56(1.16,2.12)	0.003
*No preference*	1.19(0.82,1.72)	0.35	1.30(0.89,1.89)	0.18
**Ever had sex with women**				
*No*	*Ref*		*Ref*	
*Yes*	0.76(0.57,1.02)	0.06	0.72(0.51,1.03)	0.069
**Had condomless sex with women in the last three months**				
*No*	*Ref*		*Ref*	
*Yes*	0.73(0.49,1.10)	0.14	0.79(0.50,1.24)	0.31
**Had condomless sex with men in the last three months**				
*No*	*Ref*		*Ref*	
*Yes*	1.15(0.81,1.64)	0.43	1.23(0.84,1.80)	0.28
**Ever drunk alcohol during or prior to sex in the last three months**				
*No*	*Ref*		*Ref*	
*Yes*	0.88(0.55,1.40)	0.58	0.87(0.54,1.41)	0.58
**Engaged in group sex in the last three months**				
*No*	*Ref*		*Ref*	
*Yes*	2.58(1.79,3.72)	<0.001	2.63(1.80,3.86)	<0.001
**Engaged in commercial sex in the last 12 months**				
*No*	*Ref*		*Ref*	
*Yes*	1.78(1.10,2.90)	0.019	1.86(1.13,3.06)	0.014
**Used gay apps for partners seeking in the last six months**				
*No*	*Ref*		*Ref*	
*Yes*	2.27(1.72,3.00)	<0.001	2.10(1.58,2.80)	<0.001
**HIV infection**				
*Unknown*	*Ref*		*Ref*	
*Positive*	1.53(0.86,2.72)		1.58(0.87,2.85)	0.132
*Negative*	1.57(1.21,2.04)		1.60(1.14,1.96)	0.003

Note

* Model adjusted for age (Continuous), residence (Urban or rural), education level (High school or below, college or bachelors, or masters or PhD) and monthly income (less than $250 USD, $250-300USD, $301-800USD, $801-1200USD, or more than $1200USD)

## Discussion

The increasing use of recreational drug among Chinese MSM may facilitate high-risk behaviors for HIV transmission among Chinese MSM[7]. Previous studies have shown that individuals who used recreational drug had a 2–4 times greater risk of acquiring HIV than those who did not use drugs [11,20]. Most of the literature about recreational drug use among MSM has focused on high-income contexts and its association with risk behaviors. Our study adds to the literature by recruiting participants from multiple cities throughout China, utilizing an online countrywide survery, and demonstrating the association with HIV/ STIs testing and gay apps use. Our findings indicate that individuals who use recreational drugs have a higher prevalence of risk behaviors and tend to use gay apps to find sexual partners, which has the potential to worsen the epidemic of HIV and other STIs among MSM. In addition, recreational drug users are more likely to have HIV/STI testing experience than non-users.

In the present study, even though popper use is illegal in China, approximately one-fifth of participants used poppers in the last 12 months, which is higher than the lifetime poppers use among MSM recruited in Chongqing 11.3%, Kunming 3.2%, Shenyang 1.8%, and Heilongjiang 0.3% in a 2013 study[11]. These differences may be due to the increasing use of poppers among Chinese MSM in recent years, as global data indicated that popper has become more and more population among MSM in recent years [11,14,21–23]. However, the usage rate of popper among Chinese MSM is much lower than the findings of two UK studies (53.3% in Brighton, [21], and 64.9% in London 64.9%[24]), which may be due to the fact that are legal in sex shops, clubs and bars in these cities.

Our results indicated that that poppers use was associated with using gay apps for partner seeking in the last 6 months. Gay apps utilize geospatial technology that facilitates rapid sexual partner identification and networking unbound by the constraints of time or location. [25–27] In addition, as popper uses tend to have more sexual partners[11], gay apps could facilitate the organization of private sex parties involving recreational drug use.[17] The combined use of poppers and gay apps may create a virtual risk environment for HIV transmission among Chinese MSM.

In our study, poppers users were more likely to have been tested for STIs and HIV compared with non-users, suggesting that poppers users may utilize sexual health resources at a higher rate than non- poppers users. One potential reason for this phenomenon is that after engaging in popper use and other high- risk behaviors, the popper users are more worried about their risk of HIV and other STIs infection, which linked them to HIV and other STIs testing[28–30]. Even though popper users have higher HIV and other STIs testing rates than non-users, the HIV testing rate among poppers users is still far from international guideline goals [31]. Strategies that can further promote HIV and other STIs testing are needed.

Consistent with previous literature, our study also found that recreational drug use is associated with commercial sex and group sex[23]. Unlike traditionally studied illicit drugs (e.g. heroin and marijuana), recreational drugs such as poppers can enhance sexual desire and make users have enhanced feelings of stamina and intoxicating highs[11], which may increased the likability for popper user to engage in commercial sex, group sex and having condomless anal intercourse [11,20]. Group sex often co-occurs with other high-risk behaviors that together create an environment conducive to disease transmission[32]. Previous studies showed that HIV positive MSM were more likely to have group sex and condomless anal intercourse when under the influence of recreational drugs [33,34].

This survey has several limitations. First, the MSM sample was only recruited online. Thus, the sample may fail to truly represent the actual general population. However, previous studies have confirmed the similar risk profile among online and non-online Chinese MSM based on the high Internet popularity among Chinese MSM[26]. Second, as the data was collected through self-reporting, there might be possible social desirability bias, for instance, poppers users might be reluctant to admit having used poppers, which in turn leads to underreporting [33,35]. Third, like all cross-sectional studies, selection bias due to non-response or withdraw during the study might exist in our studies [35].

Despite the above limitations, our results show that the proportion of recreational drug use, including poppers use among MSM in China is high, which has the potential to further the spread of HIV. The findings of our study stressed the need to better understand the specific characteristics of MSM who tend to use poppers and to design intervention programs targeting them. HIV interventions among MSM should not only target injecting drug use, but also reduce sexual risk behaviors. Although the Chinese government has carried out many anti-drug programs, for example, methadone maintenance treatment, these programs only target injecting drug users, and programs for MSM are limited. Chinese harm reduction programs should address the challenge of recreational drug use among MSM. HIV voluntary counseling & testing(VCT), 100% condom use program and specialist support services can be also used in Chinese MSM. Longer follow-up time for intervention to help promote behavior self-management skills may be effective for MSM risk reduction. MSM are still a hidden population in many countries, thus, new interventions methodologies (i.e. intervention delivery through social media, including gay apps) are needed.

## Supporting Information

S1 Dataset (SAV)
